# Supplementary material for: Comparative Flavor Profile of Roasted Germinated Wheat (Triticum aestivum L.) Beverages Served Hot and Cold Using Electronic Sensors Combined with Chemometric Statistical Analysis
Source: Foods. 2022 Oct 5;11(19):3099. doi: 10.3390/foods11193099 (PMC9562885; doi:10.3390/foods11193099)
Supplement: Supplementary file 1 [file foods-11-03099-s001.zip › foods-1936528-supplementary.pdf]

Table S1. Physical and nutraceutical characteristics of hot and cold wheat beverages.

Characteristics		Hot beverages			Cold beverages		
		HB_1	HB_2	HB_3	CB_1	CB_2	CB_3
Physical characteristics	Solid content (%)	0.11±0.004^{b}	0.17±0.016^{a}	0.16±0.003^{a}	0.06±0.002^{C}	0.11±0.002^{B}	0.14±0.002^{A}
	Yield (%)	0.11±0.013^{b}	0.23±0.011^{a}	0.22±0.005^{a}	0.09±0.011^{B}	0.20±0.025^{A}	0.24±0.024^{A}
Color characteristics	L*	90.86±0.09^{a}	87.95±0.64^{b}	87.18±1.27^{b}	90.21±0.01^{A}	85.99±0.09^{B}	85.08±0.01^{C}
	a*	-0.78±0.01^{c}	-0.66±0.04^{b}	-0.49±0.06^{a}	-1.16±0.01^{C}	-0.58±0.01^{B}	-0.05±0.01^{A}
	b*	6.79±0.31^{b}	11.18±1.26^{a}	10.76±1.38^{a}	14.45±0.02^{C}	26.58±0.03^{B}	29.71±0.04^{A}
	ΔE	11.43±0.25^{b}	16.48±1.31^{a}	16.76±1.84^{a}	17.51±0.02^{C}	30.06±0.06^{B}	33.26±0.03^{A}
	BI	0.08±0.01^{b}	0.12±0.01^{a}	0.11±0.00^{a}	0.21±0.00^{C}	0.36±0.00^{B}	0.46±0.00^{A}
Bioactive components	TFC (μg CE/100 mL)	3.87±0.30^{c}	11.52±0.58^{a}	8.60±0.60^{b}	10.86±0.47^{B}	25.34±1.39^{A}	32.31±1.13^{A}
	TPC (μg GAE/100 mL)	28.05±3.03^{b}	71.23±11.06^{a}	54.47±9.39^{a}	58.25±4.43^{C}	160.42±19.10^{B}	202.37±20.94^{A}
Antioxidant properties	DPPH (μM TE/100 mL)	6.15±1.70^{c}	20.39±1.39^{a}	13.12±1.08^{b}	19.56±0.87^{C}	47.25±0.62^{B}	68.43±3.41^{A}
	TEAC (mM TE/100 mL)	11.41±0.96^{c}	31.77±1.96^{a}	23.94±1.29^{b}	34.15±1.54^{C}	86.12±4.50^{B}	126.66±4.00^{A}

TFC, total flavonoid content; TPC, total phenolic content; DPPH, radical scavenging activity; TEAC, trolox equivalent antioxidant capacity; L*, lightness; a*, redness; b*, yellowness; HB_1, hot beverage 0.8 g/100 mL; HB_2, hot beverage 2 g/100 mL; HB_3, hot beverage 4 g/100 mL; CB_1, cold beverage 25 g/L; CB_2, cold beverage 50 g/L; CB_3, cold beverage 75 g/L; CE, catechin equivalent, GAE, gallic acid equivalent; TE, trolox equivalent. The different small letters indicate significant difference between hot beverages and the different capital letters indicates significant difference between cold beverages ($p < 0.05$).

Table S2. Taste properties of germinated wheat beverages (GWB) by e-tongue.

Taste properties	Hot-brewed beverages			Cold-brewed beverages		
	HB_1	HB_2	HB_3	CB_1	CB_2	CB_3
SRS_Sourness	5.87±0.05^{c}	4.23±0.05^{b}	6.10±0.08^{a}	3.07±0.12^{C}	7.67±0.05^{B}	9.03±0.05^{A}
STS_Saltiness	6.47±0.05^{b}	8.00±0.08^{b}	6.30±0.08^{a}	8.37±0.05^{A}	4.27±0.05^{B}	2.67±0.05^{C}
UMS_Umami	3.77±0.19^{c}	4.17±0.09^{b}	4.53±0.05^{a}	6.47±0.05^{C}	8.07±0.05^{B}	9.00±0.00^{A}
SWS_Sweetness	3.90±1.28^{b}	6.90±0.86^{a}	6.80±0.37^{a}	9.10±0.28^{A}	5.23±0.66^{B}	4.03±1.07^{B}
BRS_Bitterness	6.43±0.19^{b}	7.83±0.05^{a}	6.27±0.05^{b}	8.43±0.17^{A}	4.47±0.05^{B}	2.53±0.05^{C}

HB_1, hot beverage 0.8 g/100 mL; HB_2, hot beverage 2 g/100 mL; HB_3, hot beverage 4 g/100 mL; CB_1, cold beverage 25 g/L; CB_2, cold beverage 50 g/L; CB_3, cold beverage 75 g/L. Values are expressed means of three replications ± SD. The different small letters indicate significant difference between hot beverages and the different capital letters indicates significant difference between cold beverages ($p < 0.05$).
